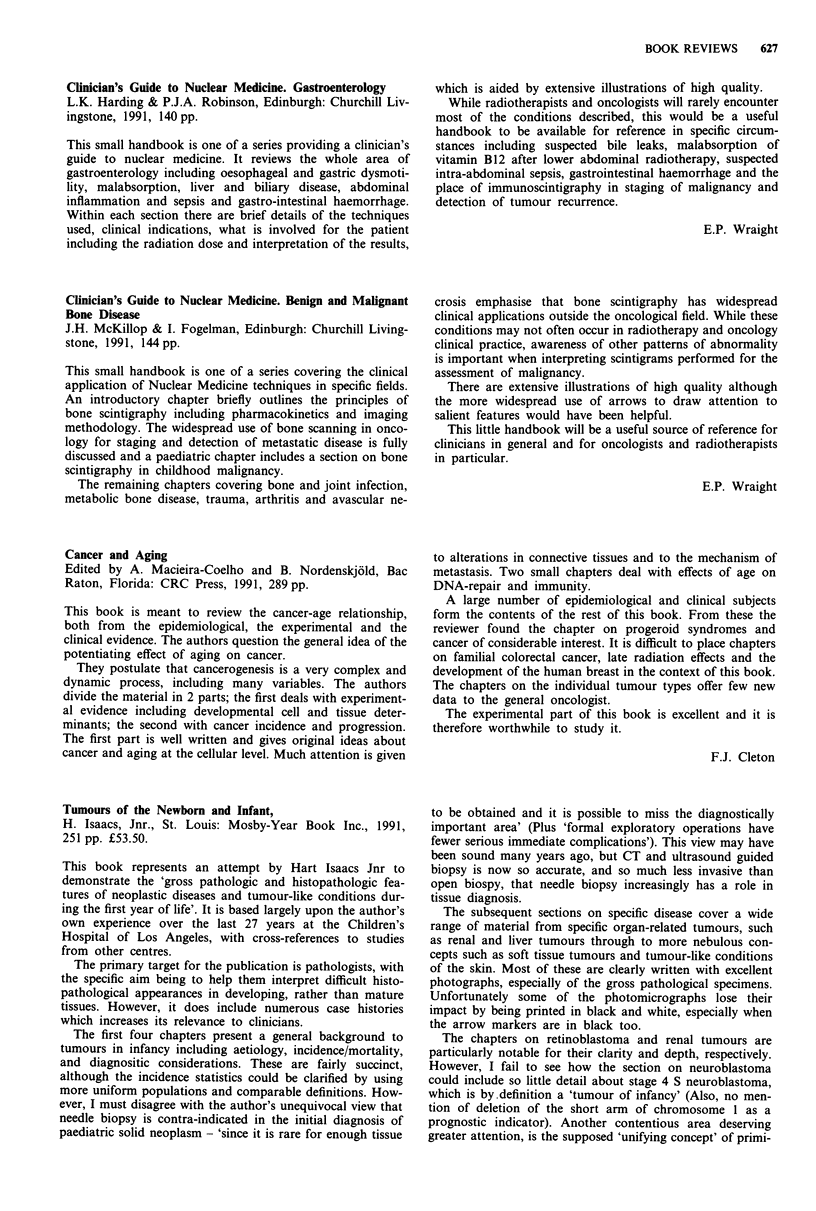# Cancer and Aging

**Published:** 1992-04

**Authors:** F.J. Cleton


**Cancer and Aging**
Edited by A. Macieira-Coelho and B. Nordenskjöld, Bac Raton, Florida: CRC Press, 1991, 289 pp.

This book is meant to review the cancer-age relationship, both from the epidemiological, the experimental and the clinical evidence. The authors question the general idea of the potentiating effect of aging on cancer.

They postulate that cancerogenesis is a very complex and dynamic process, including many variables. The authors divide the material in 2 parts; the first deals with experimental evidence including developmental cell and tissue determinants; the second with cancer incidence and progression. The first part is well written and gives original ideas about cancer and aging at the cellular level. Much attention is given to alterations in connective tissues and to the mechanism of metastasis. Two small chapters deal with effects of age on DNA-repair and immunity.

A large number of epidemiological and clinical subjects form the contents of the rest of this book. From these the reviewer found the chapter on progeroid syndromes and cancer of considerable interest. It is difficult to place chapters on familial colorectal cancer, late radiation effects and the development of the human breast in the context of this book. The chapters on the individual tumour types offer few new data to the general oncologist.

The experimental part of this book is excellent and it is therefore worthwhile to study it.

F.J. Cleton